# Supplementary figures and images for: RelocaTE2: a high resolution transposable element insertion site mapping tool for population resequencing
Source: PeerJ. 2017 Jan 26;5:e2942. doi: 10.7717/peerj.2942 (PMC5274521; doi:10.7717/peerj.2942)

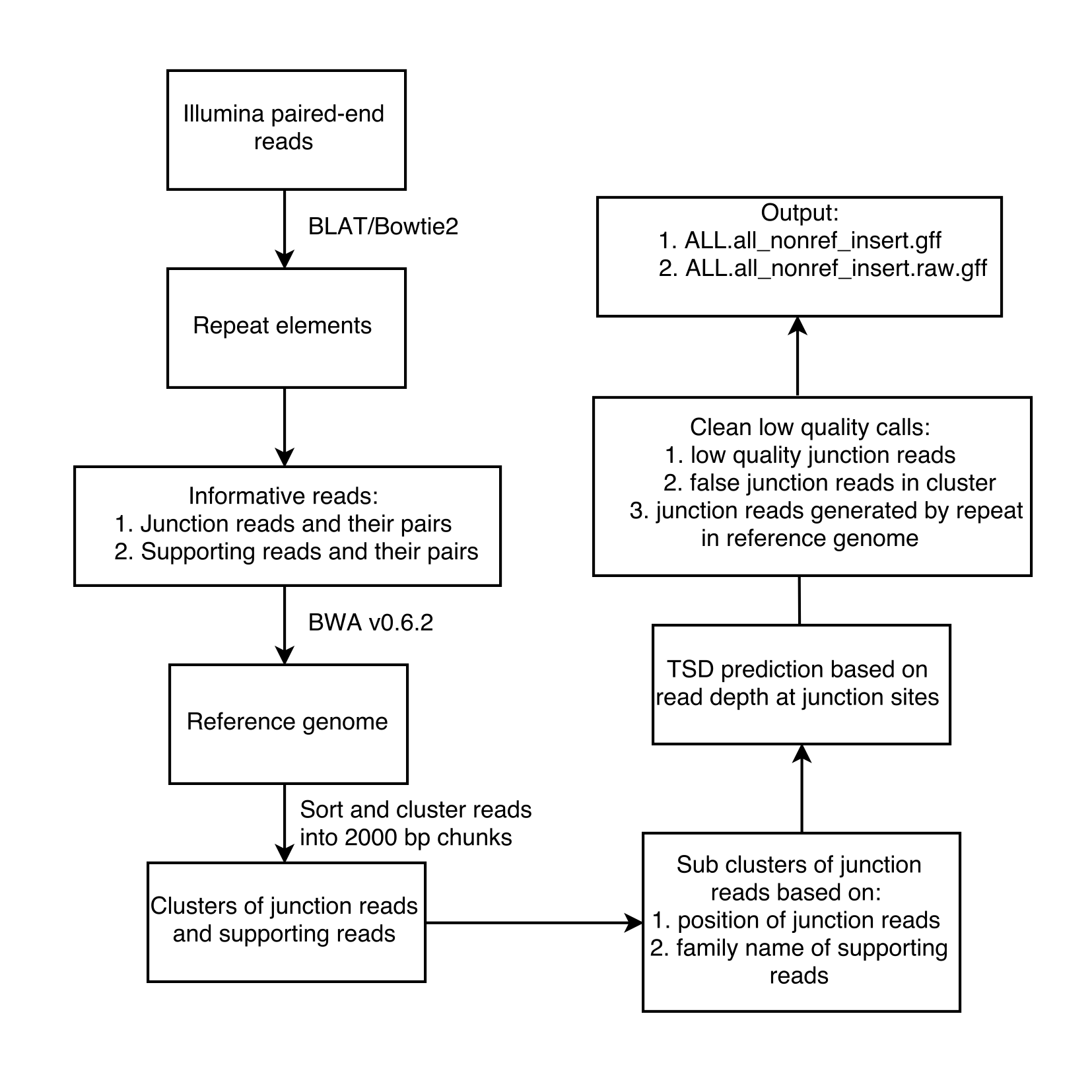

Supplement: Supplemental Information 1 — Release source code for version of RelocaTE2 described in this manuscript. [file peerj-05-2942-s001.gz › RelocaTE2-2.0.1/RelocaTE2_flowchart.png]
